# Effect of 12-*O*-tetradecanoylphorbol-13-acetate-induced psoriasis-like skin lesions on systemic inflammation and atherosclerosis in hypercholesterolaemic apolipoprotein E deficient mice

**DOI:** 10.1186/s12895-016-0046-1

**Published:** 2016-07-11

**Authors:** Marie Madsen, Peter Riis Hansen, Lars Bo Nielsen, Karsten Hartvigsen, Anders Elm Pedersen, Jan Pravsgaard Christensen, Annemarie Aarup, Tanja Xenia Pedersen

**Affiliations:** Department of Biomedical Sciences, University of Copenhagen, Copenhagen, Denmark; Department of Cardiology, Gentofte University Hospital, Gentofte, Denmark; Department of Clinical Biochemistry, Rigshospitalet, Copenhagen University Hospital, Copenhagen, Denmark; Department of International Health, Immunology, and Microbiology, University of Copenhagen, Copenhagen, Denmark; Current Address: Novo Nordisk, Gentofte, Denmark

**Keywords:** Psoriasis, Atherosclerosis, Inflammation, ApoE^−/−^ mouse, Interleukin-17, Immune cells

## Abstract

**Background:**

Risk of cardiovascular disease is increased in patients with psoriasis, but molecular mechanisms linking the two conditions have not been clearly established. Lack of appropriate animal models has hampered generation of new knowledge in this area of research and we therefore sought to develop an animal model with combined atherosclerosis and psoriasis-like skin inflammation.

**Methods:**

Topical 12-*O*-tetradecanoylphorbol-13-acetate (TPA) was applied to the ears twice per week for 8 weeks in atherosclerosis-prone apolipoprotein E deficient (ApoE^−/−^) mice.

**Results:**

TPA led to localized skin inflammation with increased epidermal thickness, infiltration of inflammatory-like cells and augmented tissue interleukin-17F levels. Systemic effects of the topical application of TPA were demonstrated by increased plasma concentration of serum amyloid A and splenic immune modulation, respectively. However, atherosclerotic plaque area and composition, and mRNA levels of several inflammatory genes in the aortic wall were not significantly affected by TPA-induced skin inflammation.

**Conclusions:**

TPA-induced psoriasis-like skin inflammation in atherosclerosis-prone ApoE^−/−^ mice evoked systemic immune-inflammatory effects, but did not affect atherogenesis. The results may question the role of psoriasis-induced inflammation in the pathogenesis of atherosclerosis in psoriasis patients.

**Electronic supplementary material:**

The online version of this article (doi:10.1186/s12895-016-0046-1) contains supplementary material, which is available to authorized users.

## Background

Psoriasis is a chronic inflammatory disease of the skin estimated to affect 2–4 % of adults in the western population, but with a varying prevalence due to factors including geography and age [1]. Epidemiological studies have demonstrated that psoriasis is associated with increased risk of cardiovascular disease, e.g., myocardial infarction, stroke, and cardiovascular death [2–6]. This association has led to recommendations for screening and aggressive management of traditional cardiovascular risk factors in psoriasis patients [7]. Indeed, cardiovascular risk factors including hyperlipidaemia, obesity, smoking, hypertension, and diabetes are also more frequently observed in psoriasis patients and the comorbidities are often underdiagnosed and undertreated [8–10].

The leading cause of cardiovascular death is atherosclerosis [11]. Like psoriasis, atherosclerosis is a chronic inflammatory disease and common immunological pathways may causally link the two diseases [10–13]. Thus, psoriasis per se may be an independent risk factor for cardiovascular disease, but it remains to be proven whether psoriasis-driven systemic inflammation accelerates atherosclerosis. The lack of appropriate models to study potential causal links between psoriasis and cardiovascular disease has hampered such investigations. Therefore, development of an animal model with psoriasis-like skin lesions and atherosclerosis would provide a valuable tool for investigations of putative shared disease mechanisms and potential new therapeutic targets aimed at both diseases. Shared immunological pathways in psoriasis and atherosclerosis include T helper cell 1 (Th1)-mediated inflammation, alterations in angiogenesis, and dysfunction of the endothelium [11]. Moreover, interleukin (IL)-17-producing cells have been found to have a key role in the pathogenesis of both psoriasis and atherosclerosis, even though the exact role of Th17 cells in atherogenesis remains debated [14].

Presently, only few studies have examined vascular changes in mice with experimentally induced psoriasis-like skin lesions and these have exclusively been performed in atherosclerosis-resistant (normocholesterolaemic) mice. Hence, the former studies have assessed vascular parameters other than established atherosclerosis, e.g., inflammatory cell infiltration, reactive oxygen species formation, endothelial dysfunction, and thrombogenicity [15, 16]. To enable investigations of potential causal links between psoriasis and atherosclerosis, we aimed to develop a mouse model combining the two diseases. The hypercholesterolaemic apolipoprotein E deficient (ApoE^−/−^) mouse is a well-established model for atherosclerosis [17, 18]. Thus, ApoE^−/−^ mice develop extensive atherosclerotic lesions detectable from approximately 10 weeks of age when on a chow diet [17]. The phorbol ester 12-*O*-tetradecanoylphorbol-13-acetate (TPA) is a protein kinase C activator, which after application to the skin induces inflammation and epidermal hyperplasia that recapitulate some of the hallmarks of psoriasis [19, 20]. To investigate whether TPA-induced skin inflammation would induce sufficient deregulation of the systemic immune-inflammatory homeostasis to affect the extent and composition of atherosclerotic plaques we therefore examined the latter after repeated applications of TPA to the ears of ApoE^−/−^ mice. Since atherosclerosis is a disease that progresses slowly, we applied TPA for 8 weeks, as has been done by others [21]. Topical TPA applications induced psoriasis-like skin lesions and unequivocal signs of increased systemic inflammation but had no effect on the development of atherosclerosis in this model.

## Methods

### Mice and topical application

Female ApoE^−/−^ mice were purchased from Taconic (Ry, Denmark), model n^o^ APO-F (B6.129P2-*ApoE*^*tm1Unc*^ N11). Two separate, but similar, studies were conducted, i.e., a pilot study with *n* = 5–7 mice/group (study 1) followed by a full-scale study with *n* = 15 mice/group (study 2). Mice had access to water and standard diet ad libitum (Altromin 1314, Brogaarden, Gentofte, Denmark) and were housed with 12 h light/dark cycles in a temperature- and humidity-controlled room at 21–23 °C at the University of Copenhagen.

At the age of 11 weeks, mice received 2 topical applications/week (20 μl/ear) of either vehicle (acetone) or TPA (Sigma-Aldrich, Brøndby, Denmark; dissolved in acetone at a 0.1 μg/μl concentration). Applications were given on both ears and the mice received 16–17 applications during a total of 8 weeks. Mice were terminated either 3–4 (study 1) or 2 (study 2) days after the last TPA application. Ear thickness was measured prior to each TPA application using a digimatic thickness gauge (Mitutoyo, Illinois, US). All measurements were performed by the same investigator. At study termination, mice were anaesthetized subcutaneously with a 0.1 ml/10 g mouse dose of either a mixture of fentanyl (0.079 mg/mL), fluanisone (2.5 mg/mL), and midazolam (1.25 mg/mL) (study 1), or a mixture of tiletamine (1.63 mg/mL), zolazepam (1.63 mg/mL), xylazin (2.61 mg/mL), and butorphanol tartrate (0.065 mg/mL) (study 2). Subsequently, blood was collected and mice were perfused with ice-cold saline.

### Skin histology

Half of an 8 mm biopsy of the right ear was prepared for histology by fixation for one week at room temperature in 10 % neutral buffered formalin (“Lillie” formaldehyde solution 4 %, Hounisen, Skanderborg, Denmark) and embedded in paraffin. Cross-sections of 4 μm were deparaffinized and rehydrated prior to staining with Mayer’s hematoxylin and eosin (Rigshospitalet, Copenhagen, Denmark), rinsing and dehydration. Digital images were obtained with a light microscope (Leica Microsystems, Ballerup, Denmark).

### Protein analysis from serum and skin samples

Blood was collected in heparinized microtubes (capiject; Terumo Medical Coorporation, Elkton, US) prior to the first TPA/acetone application (baseline sample, submandibular vein) and again at study termination (retro-orbital vein). Plasma was collected after centrifugation for 10 min at 1000 × *g* at 4 °C, aliquoted, and stored at −80 °C until use. Plasma cholesterol was measured in duplicates using the CHOD-PAP reagent from Roche (Roche Diagnostics, Denmark). For protein analyses of ear lysates, an 8 mm biopsy of the left ear was snap-frozen in liquid nitrogen. Using a tissue homogenizer (Precellys 24, Bertin Technologies, Montigny le Bretonneux, France), the biopsies were crushed in cell lysis buffer (Cell Signaling Technology, The Netherlands) containing freshly added protease inhibitors (complete protease inhibitor with Halt, Thermo Scientific, Rockford, US). Tissue lysates were collected after 15 min of centrifugation at 15,000 × *g* and total protein concentration was measured with the Pierce BCA protein assay kit (Thermo Scientific), according to the manufacturer’s instructions. Murine IL-22 and IL-17F (R&D Systems, Minneapolis, US) and serum amyloid A (SAA) (Tridelta, Kildare, Ireland) were measured by commercial ELISA according to the manufacturer’s instructions. Mouse interferon-γ (IFNγ), tumor necrosis factor- α (TNFα), keratinocyte-derived cytokine (KC), IL-1β, IL-2, IL-4, IL-5, IL-6, IL-10, IL-12p70, and total IL-12 were measured with the ProInflammatory 7-Plex and Th1/Th2 9-Plex MSD MULTI-spot Assay Systems (Meso Scale Discovery, Rockville, US) according to the manufacturer’s instructions. For each assay, a volume of 1.7–5 μl heparinized plasma or a total protein amount of 12–200 μg of ear lysate was used.

### Aortic arch atherosclerosis (*en face*) and aortic arch mRNA

The relative amount of atherosclerosis was measured *en face* in the aortic arch (from the heart to the 7^th^ rib), and the same tissue was used for RNA extraction and quantitative real-time PCR. The aortic arch (from the heart to the 7^th^ rib) was snap-frozen in liquid nitrogen. For *en face* analysis, the aortic arch was opened longitudinally, and images of the luminal surface were acquired with a digital camera connected to a dissecting microscope and analysed using the Leica IM50 software (Leica Microsystems). For mRNA analysis, total RNA was extracted from the aortic arch using TRIzol (Life Technologies, Naerum, Denmark) and examined on an Agilent 2100 Bioanalyzer (Agilent Technologies, Santa Clara, US). RNA concentration was measured using a NanoDrop 1000 Spectrophotometer (Thermo Scientific) before cDNA synthesis of 250 ng RNA/aorta using the High Capacity cDNA Reverse Transcription Kit (Life Technologies). Real-time quantitative PCR was performed on a TaqMan (Life Technologies). Primer and probe information can be found in Additional file 1.

### Aortic root histology

The apex of the heart was cut off and the remaining part fixed in Lillie’s formalin at 4 °C overnight prior to being snap-frozen in Tissue-Tek O.C.T. (Sakura Finetek, Leiden, Netherlands) in ice-cold isopentane. The aortic root was sectioned on a cryostat (Leica) at −18 to −25 °C. Ten μm sections were collected on SuperFrost Plus slides (Menzel-Gläser; Thermo Scientific) for a total of 900 μm starting from where an aortic valve cusp was first visible. The atherosclerotic plaque area was measured, where all three aortic valve cusps were visible to ensure that quantifications were performed at the same anatomical site in each mouse. Masson’s Trichrome staining was performed according to the manufacturer’s instructions (Sigma-Aldrich), and was used to detect collagen/fibrosis. Immunohistochemical staining was performed with monoclonal rat anti-mouse macrophages/monocytes (MOMA-2 MCA519, 1:500; AbD Serotec, Kidlington, UK). Corresponding antibody isotype control was run with monoclonal rat IgG2b (MAB0061, 1:500, R&D systems). For detection, we used a biotinylated secondary antibody rabbit anti-rat (E0468, 1:2000; Dako, Glostrup, Denmark). The staining procedure included blocking of endogenous peroxidase with 0.5 % H_2_O_2_, blocking of unspecific antibody binding with 2 % BSA, brown positive staining using a horse-raddish peroxidase approach (Vectastain Elite ABC kit; VectorLab) followed by diaminobenzidine (DAB+, Dako), and counterstaining with Mayer’s hematoxylin (Sigma-Aldrich). Digital photos of histological sections were acquired using a slide scanner (Pannoramic, 3DHISTECH, Budapest, Hungary or Axio Scan.Z1, Zeiss, Birkerød, Denmark), and quantified using the Visiomorph software (Visiopharm, Hørsholm, Denmark).

### Flow cytometry

Single-cell splenocyte preparations were made by gently forcing splenic tissue through a 70 μm mesh using a 3-ml syringe plunger and ice-cold Hanks Buffered Salt Solution (HBSS, Panum, Denmark). Splenocytes were pelleted at 300×*g* for 8 min, washed once in HBSS, and counted using methylene violet and the ‘Countess’ (Invitrogen). Half of the mice were euthanized in one day and the other half the following day, and each day we made a pool of splenocytes from control mice and from TPA mice. These pools were used for setup and for making ‘fluorescence minus one’ (FMO)-controls. Four different flow cytometry analyses were carried out (see Additional files 2 and 3 for more information on antibodies applied together with the corresponding representative figures for gating strategies). Cell surface staining was accomplished using standard techniques in 100 μl in V-bottom 96-well microplates (TPP Techno Plastic Products, Trasadingen, Switzerland). Briefly, 1–2 × 10^6^ splenocytes were pelleted and blocked with 50 μl FACS buffer (0.1 % sodium azide and 2 % bovine serum albumin in phosphate-buffered saline, PBS) containing FcBlock (1:100; Cat. n° 101302, BioLegend) for 5 min to block Fcγ receptors on the splenocytes. Without washing, staining antibodies were added in 50 μl FACS buffer and incubated for an additional 20 min at 4 °C in the dark. Next, splenocytes were washed, fixed with paraformaldehyde in PBS, and analysed within 24 h using LSRII flow cytometer (BD Biosciences, Albertslund, Denmark). For intracellular staining of Foxp3 (regulatory T-cells), we followed eBioscience’s protocol for staining of intracellular/nuclear proteins after cell surface markers (CD4 and CD25) had been stained using the above protocol. To assess changes in CD4^+^ helper T-cell bias due to the TPA application, we followed the manufacturer’s protocol for the Mouse Th1/Th2/Th17 Phenotyping Kit (Cat. n° 560758, BD Biosciences). In order to investigate parallel changes in CD8^+^ cytotoxic T-cell bias, an anti-CD8 antibody was added to splenocytes as described in the manufacturer’s protocol. Briefly, for individual mice, two cultures with 10 × 10^6^ splenocytes were seeded in RP-10 media (RPMI-1640 media containing 2 mM L-glutamine, 10 % heat-inactivated fetal bovine serum, 10 mM HEPES buffer, 0.1 mM non-essential amino acids, 100 U/ml penicillin, and 100 μg/ml streptomycin) containing the BD GolgiStop reagent. Splenocytes in one culture were stimulated with 50 ng/ml TPA and 1 μg/ml Ionomycin for 4 h at 37 °C, whereas the second culture was left unstimulated. Splenocytes were harvested, washed, counted, and 1.2 × 10^6^ splenocytes were fixed using BD Cytofix buffer, washed, permeabilized using BD Perm/Wash buffer, and stained using the kit’s antibody cocktail, followed by staining with the anti-CD8 antibody. Stimulated and unstimulated cells were then washed in FACS buffer prior to flow cytometric analysis.

### Statistics

Results are shown as mean ± SEM or mean ± SD for normally distributed data or median [interquartile range (IQR)] for non-normally distributed data. Differences between groups were analysed with parametric or non-parametric t-tests, and multiple t-tests with correction for multiple comparisons were used when appropriate. A p-value <0.05 was considered significant. Data were analysed using the Graphpad Prism version 6.05 (GraphPad Software, California, US).

## Results

### Long-term application of TPA induces ear swelling and local inflammation in ApoE^−/−^ mice

**Fig. 1:**
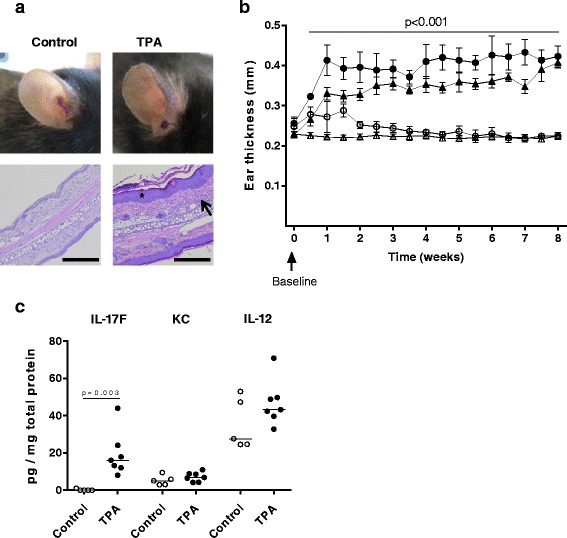
Topical 12-*O*-tetradecanoylphorbol-13-acetate (TPA) induces local skin inflammation with increased skin thickness and interleukin (IL)-17F levels. **a** Representative photos illustrating the red and scaly appearance of ears after TPA application as compared to control ears, and representative hematoxylin and eosin-stained ear cross-sections at 10× magnification. Scale bar = 200 μm. TPA led to epidermal hyperproliferation (*star*) and dermal inflammation (*arrow*). **b** Ear thickness (mm) was measured twice weekly in ApoE^−/−^ mice after TPA or vehicle application. Data from two separate, but similar studies with vehicle or TPA application on both ears were included; study 1: *n* = 5–7/group (*unfilled circle: control, filled circle*: TPA), study 2: *n* = 15/group (*unfilled triangle: control, filled triangle*: TPA). The depicted values in **b** are mean ± SD, i.e., mean value of right and left ear for each mouse. *p* < 0.001, control vs. TPA at all time points except at baseline; multiple *t*-test corrected for multiple comparisons was applied. **c** Measurement of interleukin IL-17F, IL-12, and keratinocyte-derived cytokine (KC) in ear tissue homogenates after 8 weeks of TPA or vehicle application in study 1. Data is depicted as pg cytokine per mg total protein (median values, unpaired non-parametric *t*-test)

To induce psoriasis-like skin inflammation, hypercholesterolaemic ApoE^−/−^ mice received twice weekly topical applications on both ears of either TPA or vehicle (control), for 8 weeks. TPA led to a skin reaction characterized by scaly skin and redness (Fig. 1a), and by a marked increase in ear thickness throughout the application period (*p* < 0.001 at all time points after baseline, TPA vs. control, Fig. 1b). The ear thickness in control mice was not affected by vehicle application. Histological examination of hematoxylin and eosin-stained ear cross-sections revealed that TPA induced epidermal thickening and local inflammation as assessed by the presence of inflammatory cells in the dermis (Fig. 1a). To investigate whether the TPA-induced histological features were accompanied by changes in local levels of inflammatory mediators, we measured protein levels of selected cytokines in ear lysates. Levels of IL-17F were significantly higher in ear lysates from TPA-treated mice as compared to those from control mice (16.2 [12.1–24.1] pg/mg total protein vs. 0 [0.0–0.5] pg/mg total protein, *p* = 0.003, Fig. 1c), indicating that topical TPA application induced a local immune response with infiltration of IL-17F producing cells. We found no difference in protein levels of IL-12 and KC. Protein levels of the cytokines IL-1β, −2, −4, −5, −6, −10, −12p70, −22, and IFNγ, and TNFα were below the ELISA detection limits in all ear lysates.

### Topical TPA application elicits systemic inflammatory modulations

**Fig. 2:**
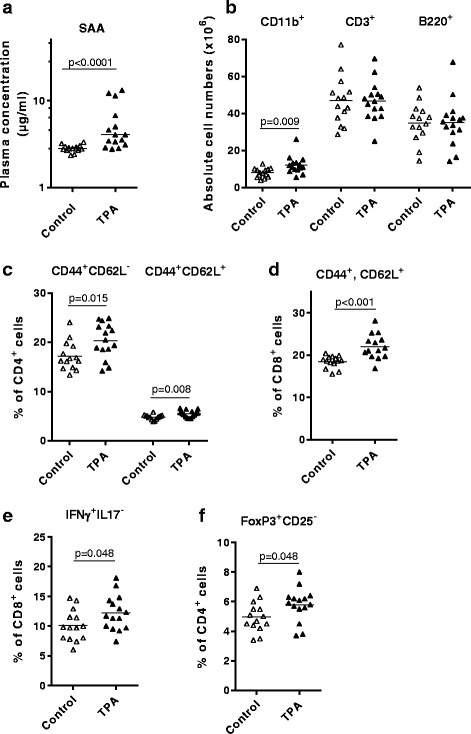
Topical 12-*O*-tetradecanoylphorbol-13-acetate (TPA) application induces systemic inflammation. **a** Serum amyloid A (SAA) levels (μg/ml) measured in plasma after 8 weeks of topical application on both ears with either TPA or vehicle (control). Note: the y-axis is displayed as a log10 scale. Values are depicted as median and statistical differences analysed by non-parametric *t*-test. **b**-**f** Spleen flow cytometry data. Symbols and horizontal bars indicate individual mice and group averages, respectively, and parametric t-tests were used to detect statistical differences. **b** Levels of CD11b^+^ cells, T-cells (CD3^+^), and B-cells (B220^+^). **c** Effector and memory CD4^+^ T-cells (CD44^+^CD62L^−^ and CD44^+^CD62L^+^ cells, respectively), and **d** memory CD8^+^ T-cells (CD44^+^CD62L^+^ cells). **e** Tc1 cells (IFNγ^+^IL17^−^). **f** Regulatory T-cells (Foxp3^+^CD25^−^). *Unfilled and filled triangles* represent control and TPA mice, respectively, from study 2. *N* = 14–15 mice/group; one control mouse was omitted from flow cytometry data due to abnormal values (i.e., above 3xSD)

To investigate whether topical application of TPA would induce not only a local immune response, but also systemic effects, we measured plasma levels of SAA and inflammatory cytokines (IL-1β, −2, −4, −5, −6, −10, and -12p70, and IFNγ, and TNFα), and performed flow cytometry of spleens from TPA and control mice. Plasma SAA levels were higher in TPA-treated vs. control mice (4.1 [3.1–6.7] μg/ml vs. 2.8 [2.7–3.0] μg/ml, *p* < 0.0001, Fig. 2a), whereas the other measured cytokines either were below ELISA detection limits, or showed no difference between the two groups (data not shown). TPA application caused larger spleens compared to vehicle application (5.4 ± 0.2 vs. 4.6 ± 0.2 mg wet weight/body weight, *p* = 0.0039); however, this difference was not reflected in absolute splenocyte numbers (105 ± 8 × 10^6^ vs. 98 ± 7 × 10^6^ cells, p > 0.05). Flow cytometry analyses revealed a significantly higher amount of CD11b^+^ cells in spleens from TPA-treated mice compared to control mice (12.0 ± 1.2 vs. 7.9 ± 0.7 × 10^6^ cells, *p* = 0.009, Fig. 2b). In mouse spleen, CD11b is expressed primarily by inflammatory monocytes, macrophages, neutrophils, and some subpopulations of dendritic cells [22]. Additional flow cytometry analyses of the spleens did not reveal differences in cytotoxic (CD8^+^) or helper (CD4^+^) T-cell populations (data not shown). However, detailed analyses of activated CD4^+^ and CD8^+^ T-cell populations, based on expression pattern of CD62L and CD44, revealed significantly expanded effector (CD44^+^CD62L^−^) and memory (CD44^+^CD62L^+^) CD4^+^ T-cell populations in the TPA mice compared to control mice (Fig. 2c). In addition, the memory (CD44^+^CD62L^+^) CD8^+^ T-cell population was also expanded in the TPA mice (Fig. 2d). There were corresponding reductions of naïve CD4^+^ and CD8^+^ T-cell populations (data not shown). Using TPA/ionomycin-stimulation of splenocytes, we detected similar expression of intracellular IFN-γ, IL-4, and IL-17 (Th1, Th2, and Th17 signature cytokines, respectively) in CD4^+^ cells from TPA and control mice (data not shown). However, in the CD8^+^ cells, we found a significantly higher percentage of IFN-γ expression (Tc1-cells) in TPA mice (Fig. 2e). There was a corresponding reduction of uncommitted CD8^+^ cells, but no differences in IL-4 and IL-17 expression in the CD8^+^ cells (data not shown). In a separate analysis, we found significantly elevated percentages of splenic CD4^+^Foxp3^+^CD25^−^ regulatory T-cells (Tregs) in TPA mice compared to control mice (Fig. 2f). There were no differences in natural CD4^+^Foxp3^+^CD25^+^ Tregs or activated CD4^+^CD25^+^Foxp3^−^ T-cells (data not shown).

### Topical application of TPA does not accelerate atherosclerosis in ApoE^−/−^ mice

**Fig. 3:**
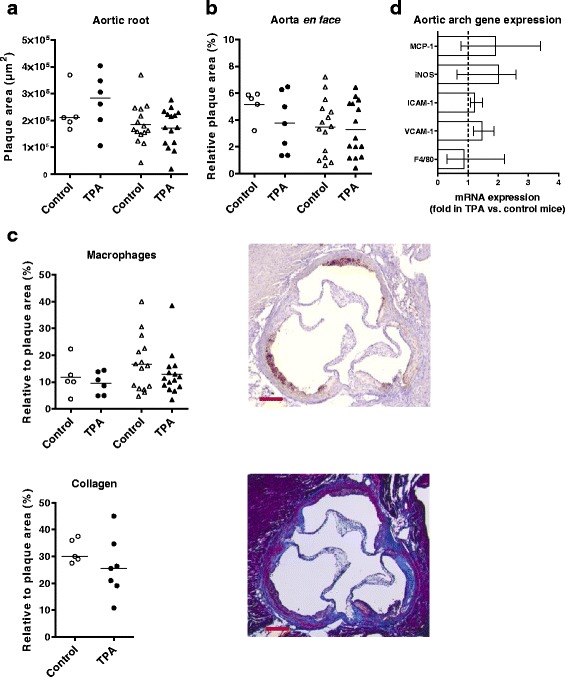
Topical 12-*O*-tetradecanoylphorbol-13-acetate (TPA) application does not affect atherogenesis in apolipoprotein E deficient (ApoE^−/−^) mice. Plaque areas measured in control and TPA mice in: **a** cross sections of the aortic root (μm^2^), and **b** the aortic arch *en face* (% of the aortic arch area); data represent mean values, parametric *t*-test. In **a**, the number of sections quantified were *n* = 4–7/mouse in study 1, and *n* = 1–4/mouse in study 2. **c** Quantification of the level of macrophages and monocytes (MOMA-2, *brown*) and collagen content (Trichrome, *blue*) in aortic root plaque (depicted as % of the total plaque area in the aortic root, mean values, parametric t-tests). Data from study 1 (*n* = 5–7/group) are shown as follows: control: *unfilled circle*; TPA: *filled circle*, and from study 2 (*n* = 10–15/group) as control: unfilled triangle; TPA: filled triangle. Also shown representative photos of the two aortic root stainings, with scale bar = 200 μm. **d** mRNA levels of the macrophage marker F4/80, vascular adhesion molecule 1 (VCAM-1), intercellular adhesion molecule 1 (ICAM-1), inducible nitric oxide synthase (iNOS), and monocyte chemoattractant protein 1 (MCP-1) in the aortic arch as measured by real-time quantitative PCR. The expression levels were normalized to the housekeeping gene glycealdehyde-3-phosphate-dehydrogenase (GAPDH). Subsequently, fold expression in TPA mice relative to control mice was calculated and depicted (control mice set to 1 and depicted as a dotted line). Ten mice/group from study 2 were randomly selected for this analysis. Results are shown as median (IQR), and statistical differences were analysed with non-parametric *t*-test

Atherosclerotic plaque area in the aortic root as well as in the aorta *en face* was similar in TPA-treated and control mice (Figs. 3a and b). Also, we found no differences in plasma cholesterol levels (Additional file 4) or in the composition of the plaques in the aortic root, as assessed by histological staining for macrophages and collagen (Fig. 3c). To investigate whether more subtle inflammatory changes had occurred in the arterial wall, we measured aortic arch mRNA expression of several genes involved in atherogenesis, i.e., macrophage markers (F4/80, murine monocyte chemoattractant protein-1 [MCP-1]), adhesion molecules (intercellular adhesion molecule 1 [ICAM-1], vascular cell adhesion molecule 1 [VCAM-1]), and inducible nitric oxide synthase [iNOS]). None of these genes were differentially expressed between TPA and control mice (Fig. 3d).

## Discussion

In the present study, we demonstrated that long-term topical TPA application in hypercholesterolaemic ApoE^−/−^ mice induced skin inflammation with psoriasis-like features, i.e., epidermal thickening and increased local IL-17F levels in the skin, presumably reflecting skin infiltration of IL-17F-producing immune cells. TPA application also led to systemic effects, as identified by higher plasma levels of SAA and splenic weight, and altered splenic cellular populations. These systemic immunomodulatory effects of TPA-induced dermatitis, however, did not affect the area and composition of atherosclerotic plaques, and had no effect on aortic expression of a range of inflammatory genes.

Despite strong epidemiological associations, it is unclear which mechanisms mediate the increased risk of cardiovascular disease in patients with psoriasis. In patients with psoriasis, clinical data from treatment studies with antibodies against TNFα and IL-17, suggest that Th1 and Th17 cells play a significant role in development and progression of psoriasis [23]. Moreover, IL-17A, IL-17C, and IL-17F protein levels are increased in psoriatic lesions in humans as well as in some mouse models of psoriasis, e.g., after TPA application in transgenic mice with skin-specific expression of vascular endothelial growth factor, and in the imiquimod (a toll-like receptor 7 and 8 ligand and potent immune stimulator) model [24, 25]. KC, the proposed murine functional analogue of human IL-8, is a pro-inflammatory chemokine that has also been shown to play a role in human psoriasis pathogenesis [26]. In our study, we found higher levels of IL-17F, and similar levels of KC in TPA mice compared to vehicle-treated mice. Thus, our data indicate that in ApoE^−/−^ mice, the TPA-induced skin lesions involve IL-17F-producing cells, but our negative results for the range of other investigated inflammatory cytokines in the ear lysates suggest that important differences exist between the immuno-inflammatory mechanisms in human psoriasis compared to the TPA model.

To assess whether the TPA-induced cutaneous lesions affected the mice systemically, we measured plasma levels of SAA and selected cytokines. SAA is a circulating acute phase protein in humans and in mice (where expression of C-reactive protein [CRP] is negligible) and hepatic SAA production is stimulated by IL-1, IL-6, and TNFα [27]. Plasma levels of SAA and CRP have been reported to be up-regulated in psoriasis patients [28, 29]. In our study, plasma levels of SAA were significantly higher in ApoE^−/−^ mice with TPA-induced skin inflammation as compared to vehicle-treated mice. None of the other investigated cytokines were increased in plasma, and most were below detection limit of our assays. These findings indicate that topical application of TPA induced a relatively low-grade systemic inflammation which may be comparable to the relatively modest increases of circulating levels of inflammatory markers that have been found in patients with psoriasis [30]. Flow cytometric analyses of the spleen revealed that topical application of TPA increased the number of CD11b^+^ cells and also caused more subtle changes with expanded populations of effector (CD44^+^CD62L^−^) CD4^+^ T-cells and memory (CD44^+^CD62L^+^) CD4^+^ and CD8^+^ T-cells, together with a relative increase in Tc1-cells and Tregs. Interestingly, hyper-activated effector T-cells and a considerable number of Tregs are present in psoriatic skin lesions, where the ability of Tregs to suppress inflammation may be diminished by mechanisms dependent on IL-6, but the relevance of our findings to these abnormalities in patients with psoriasis are unclear at present [31, 32]. Notwithstanding, the immunomodulatory effects on spleen cell populations after TPA application in the present study did not lead to increased atherosclerosis and it is possible that the strength and specificity of these effects were insufficient to affect atherogenesis in this model.

Only very few studies have been published that examined mechanisms by which psoriatic skin lesions may influence vascular biology. The KCTie2 doxycycline-repressible murine model of psoriasis with transgenic expression in keratinocytes of the angiopoietin receptor Tie2 was reported to develop systemic inflammation and aortic root vasculitis in one third of the mice at 12 months of age and these mice had shortened time to occlusive thrombus formation in a model of photochemical carotid artery thrombosis [16]. Very recently, results from a K14-IL-17A^ind/+^ mouse model with keratinocyte overexpression of IL-17A were published and these animals developed very severe psoriasis-like skin lesions and displayed increased vascular oxidative stress, endothelial dysfunction, hypertension, left ventricle hypertrophy, and markedly reduced survival as compared to controls [15]. In both studies, the psoriatic skin inflammation therefore significantly affected the vascular system, but it was not possible to assess the effect of skin inflammation on atherogenesis since these mouse models were normocholesterolaemic and thus resistant to development of atherosclerosis. In our study, we used the hypercholesterolaemic atherosclerosis-prone ApoE^−/−^ mouse, and atherosclerosis was measured both in the aorta *en face* and in aortic root cross sections. We found no evidence that the TPA-induced skin inflammation and systemic inflammatory changes significantly influenced atherosclerotic plaque size, plaque composition or aortic arch mRNA levels of inflammatory mediators. Of note, similar results have previously been obtained in ApoE^−/−^ mice with chronic dermatitis induced by croton-oil, the compound from which TPA was originally isolated. However, in that study, mice were challenged only once per 4 weeks, with 8 applications in total, there was no evidence of sustained systemic inflammation, and atherosclerosis was assessed exclusively by aortic *en face* lesion area [33]. Our results add considerably to these earlier data by showing that although experimental induction of psoriasis-like skin lesions led to systemic inflammation, atherosclerosis in the ApoE^−/−^ model was not significantly affected. This finding should be interpreted in light of the limitations of our study, e.g., the inflammatory status of the ApoE^−/−^ model may represent an overwhelming stimulus that abrogates the influence of skin lesions, the immuno-stimulatory effects of TPA are unlikely to reproduce all abnormalities found in psoriasis, and the relatively small area of psoriasis-like skin lesions in the model, where the ears measure approximately 1 cm^2^ on each side thus representing about 6 % of total mouse body surface area [34]. Indeed, a maximum severity psoriasis lesion with 6 % of total body area involvement corresponds to a Psoriasis Area Severity Index (PASI, the most widely used tool to clinically assess psoriasis severity) of 5, compatible with mild-to-moderate disease [35]. On the other hand, we found that these skin lesions elicited unequivocal signs of increased systemic inflammation and it is notable that even mild psoriasis has been associated with increased risk of myocardial infarction and stroke [2, 3]. If TPA had also been applied to the back skin, the systemic inflammatory response might have been stronger. However, we decided against this procedure, since the ApoE^−/−^ mouse is on a C57Bl6/j background and has patches on the back skin, where the cycle of hair follicles is not synchronized after the age of approximately 10 weeks. When analysing effects of TPA application over 8 weeks hereafter, this ‘patching’ makes is impossible to compare skin lesions on the same anatomical site in different mice. Also, topical application of imiquimod has been suggested to be a more representative model of psoriasis [25]. However, all animal models of psoriasis carry inherent limitations and although keratinocyte signal transduction after stimulation with TPA or imiquimod shows similarities, e.g., with involvement of nuclear factor kappa B (NF-kB) and signal transducer and activator of transcription 3 (STAT3) pathways, important differences between imiquimod-induced skin inflammation and psoriatic plaques were recently demonstrated [36, 37].

## Conclusions

In summary, we have investigated a new mouse model that potentially allows for long-term studies of effects of psoriasis-like skin lesions in hypercholesterolaemic mice. Our data suggest that in ApoE^−/−^ mice, TPA-induced psoriasis-like skin lesions lead to both local and systemic inflammation, but despite these effects, we found no alteration in atherosclerotic plaque development. Thus, additional animal models are needed to examine the hypothesis that psoriasis can promote cardiovascular disease.

## Abbreviations

ApoE^−/−^, apolipoprotein E deficient; CRP, C-reactive protein; DAB+, diaminobenzidine; FMO, fluorescence minus one; GAPDH, glycealdehyde-3-phosphate-dehydrogenase; HBSS, Hanks Buffered Salt Solution; ICAM-1, intercellular adhesion molecule 1; IFNγ, interferon-γ; IL, interleukin; iNOS, inducible nitric oxide synthase; IQR, interquartile range; KC, keratinocyte-derived cytokine; MCP-1, murine monocyte chemoattractant protein-1; NF-kB, nuclear factor kappa B; PASI, Psoriasis Area Severity Index; SAA, serum amyloid A; STAT3, signal transducer and activator of transcription 3; Th1, T helper cell 1; TNFα, tumor necrosis factor- α; TPA, 12-*O*-tetradecanoylphorbol-13-acetate; Tregs, regulatory T-cells; VCAM-1, vascular cell adhesion molecule 1

## Additional files

Additional file 1: Primers used for quantitative real-time PCR. (DOCX 14 kb) Additional file 2: Antibodies used for flow cytometry. (DOCX 16 kb) Additional file 3: Flow cytometry gating strategy. A. Gating strategy in Fig. 2b: Detection of CD3^+^, B220^+^, and CD11b^+^ cells. B. Gating strategy in Fig. 2c and d: Detection of effector and memory CD4^+^ and CD8^+^ T-cells. C. Gating strategy in Fig. 2e: Mouse Th1/Th2/Th17 Phenotyping Kit plus CD8 staining. D. Gating strategy Fig. 2f: Detection of regulatory T-cells (Tregs). (PPTX 601 kb) Additional file 4: Mouse body weight and plasma cholesterol. Mean ± SEM, unpaired parametric test control vs. 12-*O*-tetradecanoylphorbol-13-acetate (TPA) mice at baseline and termination in both studies (no statistical significant differences were found between the two groups of mice). (DOCX 14 kb)
