# Supplementary material for: Effect of 12-O-tetradecanoylphorbol-13-acetate-induced psoriasis-like skin lesions on systemic inflammation and atherosclerosis in hypercholesterolaemic apolipoprotein E deficient mice
Source: BMC Dermatol. 2016 Jul 11;16:9. doi: 10.1186/s12895-016-0046-1 (PMC4940745; doi:10.1186/s12895-016-0046-1)
Supplement: Additional file 1: — Primers used for quantitative real-time PCR. (DOCX 14 kb) [file 12895_2016_46_MOESM1_ESM.docx]

**Additional file 1. Primers used for quantitative real-time PCR.**

|  | **Forward primer** | **Reverse primer** |
| --- | --- | --- |
| Glyceraldehyde 3-Phosphate Dehydrogenase (GAPDH) | 5’-GTGGTTCACACCCATCACAA-‘3 | 5’-GGTGCTGAGTATGTCGTGGA -’3 |
| Murine monocyte chemoattractant protein-1 (MCP-1) | 5’-AGGTCCCTGTCATGCTTCTG-’3 | 5’-TCTGGACCATTTCCTTCTTG-’3 |
| Inducible nitric oxide synthase (iNOS) | 5’-ACTGGGTGAACTCCAAGGTG’-3 | 5’-GCATCCCAAGTACGAGTGGT-’3 |
| Intercellular adhesion molecule 1 (ICAM-1) | 5’-ATGCCGACCCAGGAGAGCACAA-’3 | 5’-TCGACGCCGCTCAGAAGAACCA-’3 |
| Vascular cell adhesion molecule 1 (VCAM-1) | 5’-CTTCATCCCCACCATTGAAG-’3 | 5’-TGAGCAGGTCAGGTTCACAG-’3 |
| For murine F4/80, a probe was used (Mm00802529_m1, Life Technologies). |  |  |
