# Supplementary material for: Effect of 12-O-tetradecanoylphorbol-13-acetate-induced psoriasis-like skin lesions on systemic inflammation and atherosclerosis in hypercholesterolaemic apolipoprotein E deficient mice
Source: BMC Dermatol. 2016 Jul 11;16:9. doi: 10.1186/s12895-016-0046-1 (PMC4940745; doi:10.1186/s12895-016-0046-1)
Supplement: Additional file 2: — Antibodies used for flow cytometry. (DOCX 16 kb) [file 12895_2016_46_MOESM2_ESM.docx]

**Additional file 2. Antibodies used for flow cytometry.**

| **Target** | **Label** |  | **Clone** | **Company** | **Cat. n°** |
| --- | --- | --- | --- | --- | --- |
|  | | | | | |
| Fig. 2b | | | | | |
| **B220** | PerCP-Cy5.5 |  | RA3-6B2 | BioLegend | 103236 |
| **CD11b** | APC |  | M1/70 | BioLegend | 101212 |
| **CD3** | FITC |  | 145-2c11 | BioLegend | 100305 |
|  | | | | | |
| *FMO-control without CD11b* | | | | | |
|  |  |  |  |  |  |
| Fig. 2c and 2d | | | | | |
| **CD4** | PerCP-Cy5.5 |  | GK1.5 | BioLegend | 100434 |
| **CD8a** | APC |  | 53.6.7 | BioLegend | 100712 |
| **CD62L** | PE |  | MEL-14 | BioLegend | 104408 |
| **CD44** | FITC |  | IM7 | BioLegend | 103006 |
|  | | | | | |
| *FMO-control without CD62L*  *FMO-control without CD44* | | | | | |
|  |  |  |  |  |  |
| Fig. 2e | | | | | |
| **CD4** | PerCP-Cy5.5 |  | RM4-5 | BD Biosciences | 560758 (Mouse Th1/Th2/Th17  Phenotyping Kit) |
| **IL-17A** | PE |  | 53.6.7 |  |  |
| **IFNγ** | FITC |  | PC61 |  |  |
| **IL-4** | APC |  | FJK-16s |  |  |
|  |  |  |  |  |  |
| **CD8a** | PE-Cy7 |  | 56-6.7 | BioLegend | 100722 |
|  |  |  |  |  |  |
| Fig. 2f | | | | | |
| **CD4** | PerCP-Cy5.5 |  | GK1.5 | BioLegend | 100434 |
| **CD25** | PE |  | PC61 | BioLegend | 102008 |
| **Foxp3** | Alexa 488 |  | FJK-16s | eBioscience | 53-5773-82 |
|  | | | |  |  |
| Foxp3 staining buffer set | | | | eBioscience | 00-5523-00 |
| FACS staining buffer | | | | eBioscience | 00-4222-26 |
|  | | | |  |  |
| *FMO-control without CD25*  *FMO-control without Foxp3* | | | | | |
