# Supplementary material for: Effect of 12-O-tetradecanoylphorbol-13-acetate-induced psoriasis-like skin lesions on systemic inflammation and atherosclerosis in hypercholesterolaemic apolipoprotein E deficient mice
Source: BMC Dermatol. 2016 Jul 11;16:9. doi: 10.1186/s12895-016-0046-1 (PMC4940745; doi:10.1186/s12895-016-0046-1)
Supplement: Additional file 3: — Flow cytometry gating strategy. A. Gating strategy in Fig. 2b: Detection of CD3+, B220+, and CD11b+ cells. B. Gating strategy in Fig. 2c and d: Detection of effector and memory CD4+ and CD8+ T-cells. C. Gating strategy in Fig. 2e: Mouse Th1/Th2/Th17 Phenotyping Kit plus CD8 staining. D. Gating strategy Fig. 2f: Detection of regulatory T-cells (Tregs). (PPTX 601 kb) [file 12895_2016_46_MOESM3_ESM.pptx]

## Slide 1
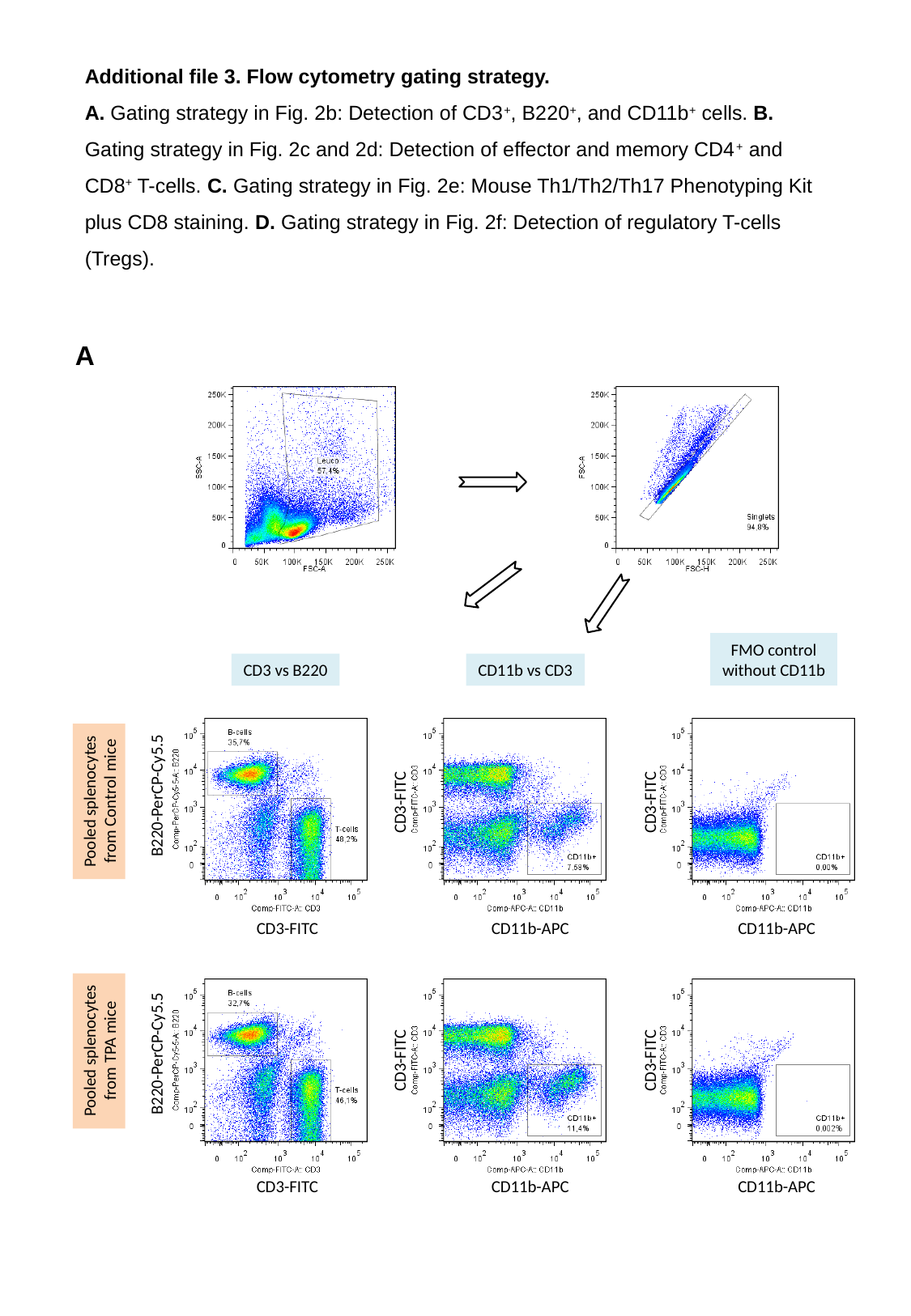

Additional file 3. Flow cytometry gating strategy.
A. Gating strategy in Fig. 2b: Detection of CD3+, B220+, and CD11b+ cells. B. Gating strategy in Fig. 2c and 2d: Detection of effector and memory CD4+ and CD8+ T-cells. C. Gating strategy in Fig. 2e: Mouse Th1/Th2/Th17 Phenotyping Kit plus CD8 staining. D. Gating strategy in Fig. 2f: Detection of regulatory T-cells (Tregs).
A
FMO control
without CD11b
CD3 vs B220
CD11b vs CD3
Pooled splenocytes
from Control mice
B220-PerCP-Cy5.5
CD3-FITC
CD3-FITC
CD3-FITC
CD11b-APC
CD11b-APC
Pooled splenocytes
from TPA mice
B220-PerCP-Cy5.5
CD3-FITC
CD3-FITC
CD3-FITC
CD11b-APC
CD11b-APC

## Slide 2
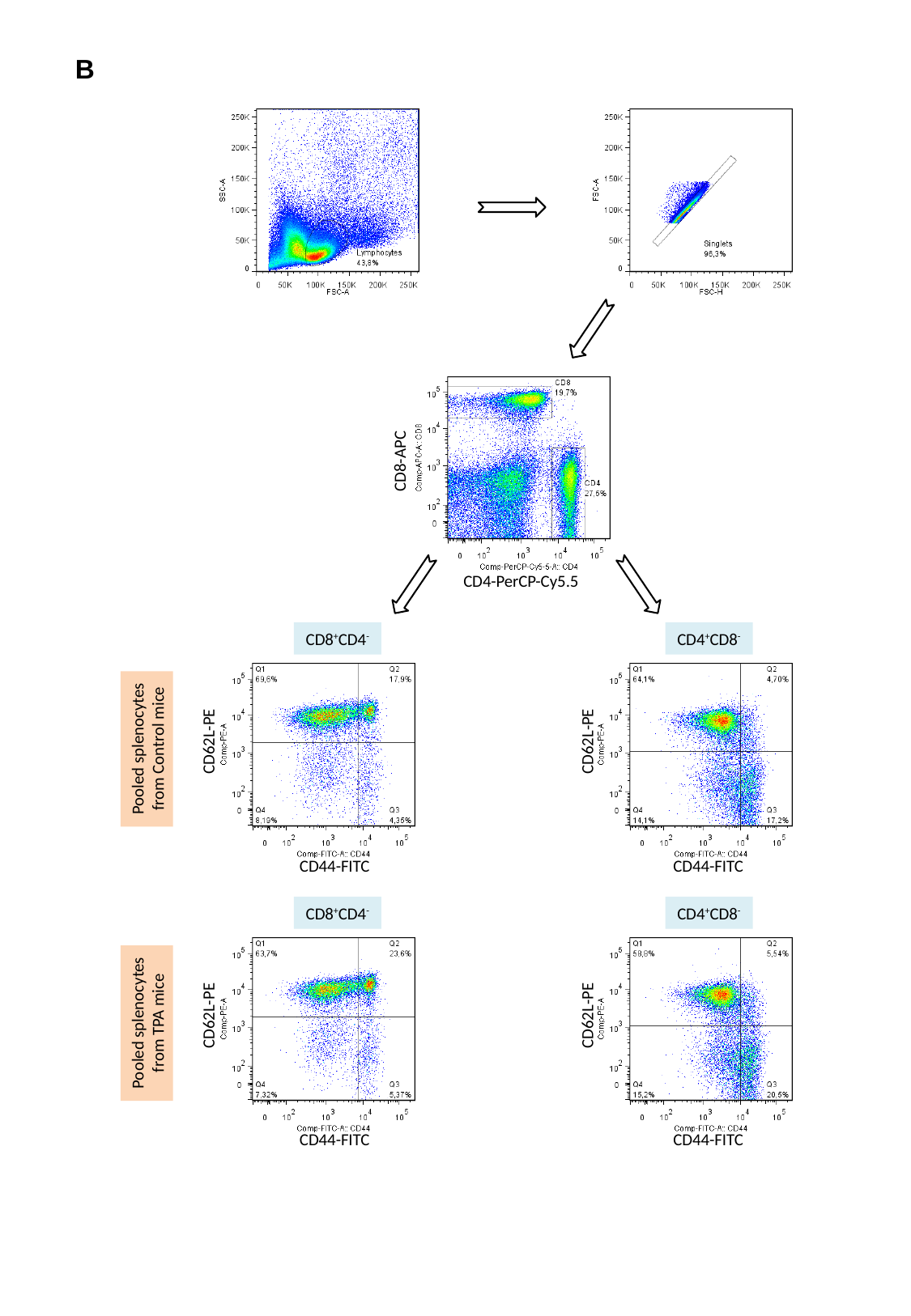

B
CD8-APC
CD4-PerCP-Cy5.5
CD8+CD4-
CD4+CD8-
Pooled splenocytes
from Control mice
CD62L-PE
CD62L-PE
CD44-FITC
CD44-FITC
CD8+CD4-
CD4+CD8-
Pooled splenocytes
from TPA mice
CD62L-PE
CD62L-PE
CD44-FITC
CD44-FITC

## Slide 3
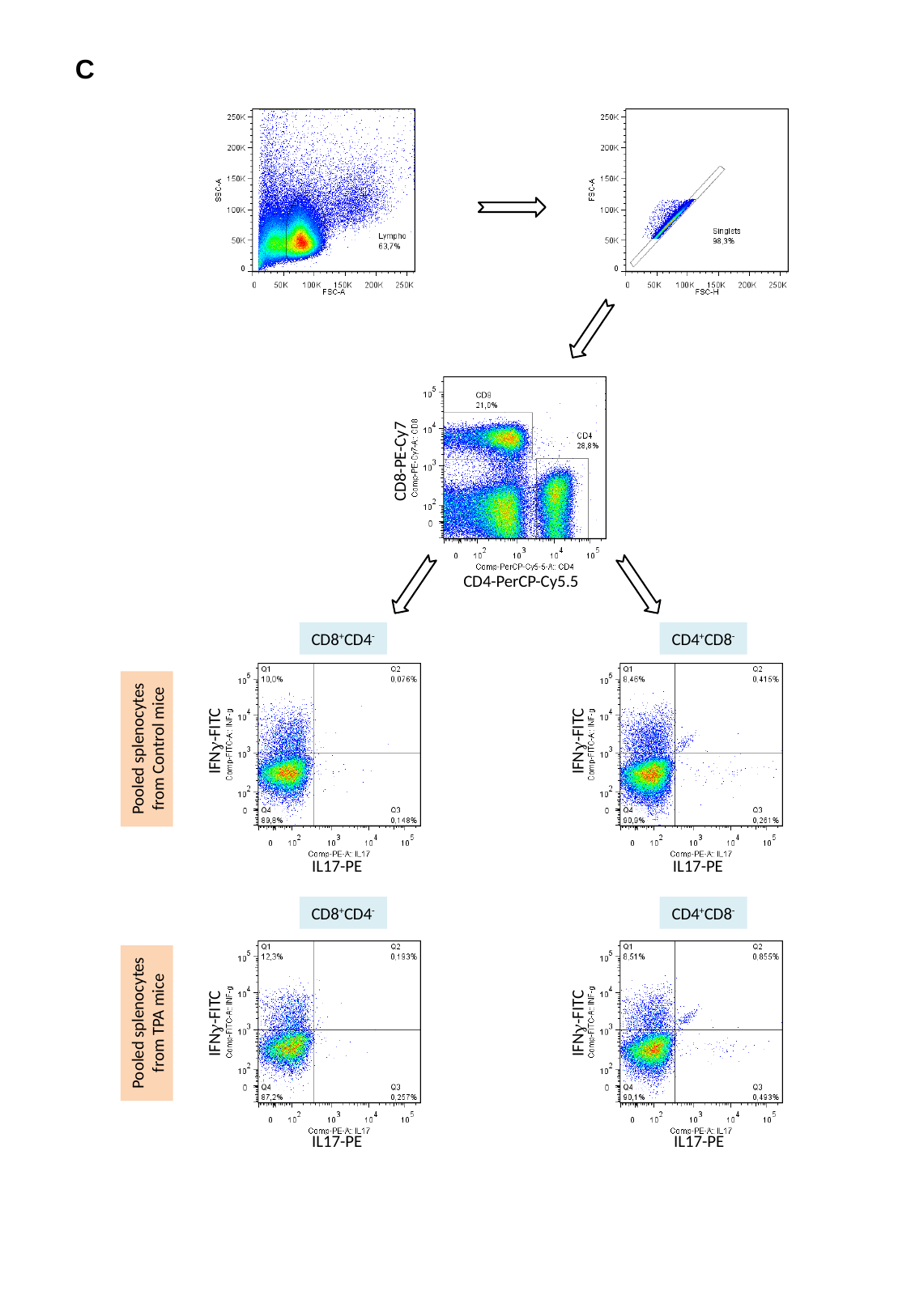

C
CD8-PE-Cy7
CD4-PerCP-Cy5.5
CD8+CD4-
CD4+CD8-
IFNg-FITC
IFNg-FITC
IL17-PE
IL17-PE
CD8+CD4-
CD4+CD8-
IFNg-FITC
IFNg-FITC
IL17-PE
IL17-PE
Pooled splenocytes
from Control mice
Pooled splenocytes
from TPA mice

## Slide 4
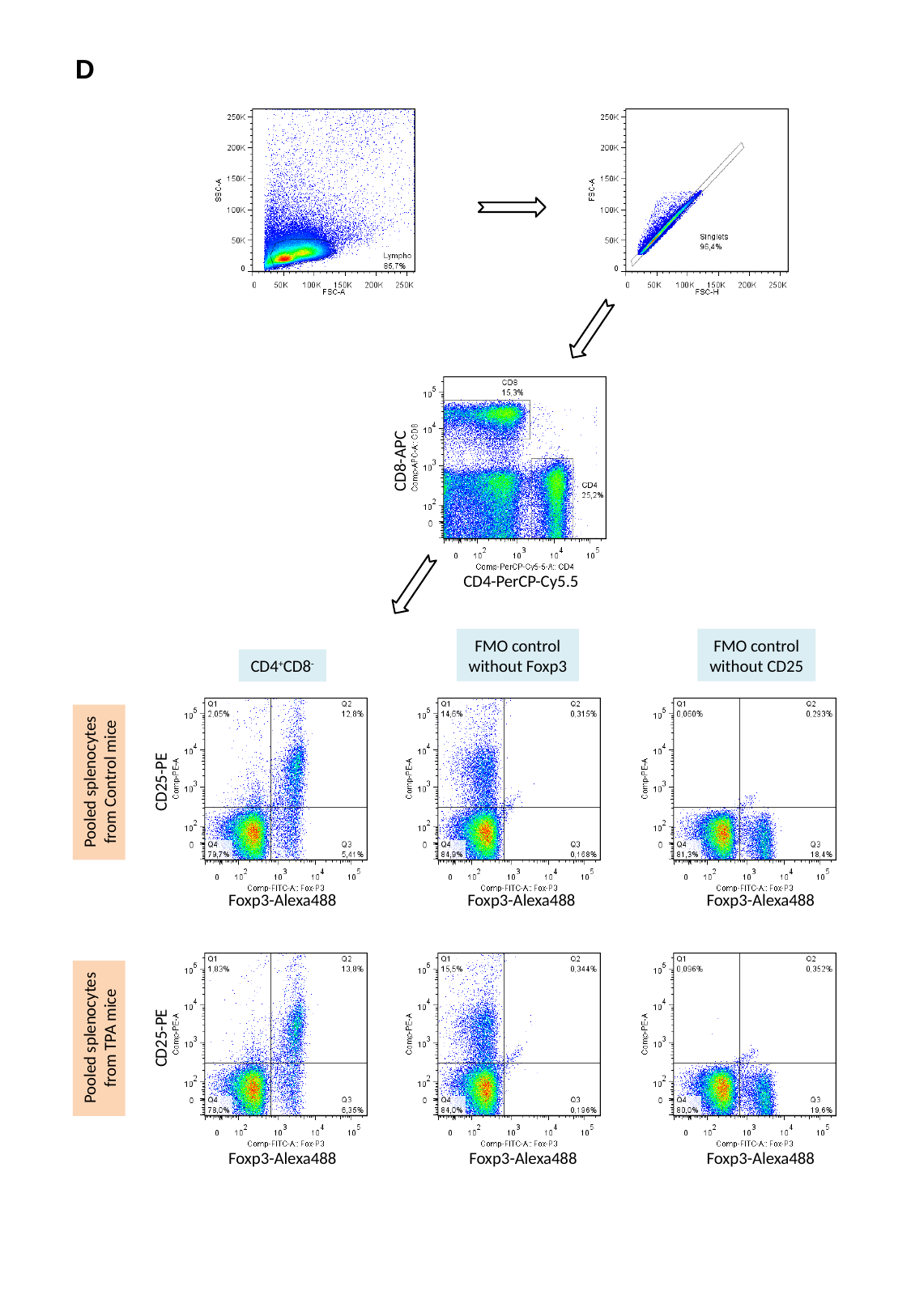

D
CD8-APC
CD4-PerCP-Cy5.5
FMO control
without Foxp3
FMO control
without CD25
CD4+CD8-
Pooled splenocytes
from Control mice
CD25-PE
Foxp3-Alexa488
Foxp3-Alexa488
Foxp3-Alexa488
Pooled splenocytes
from TPA mice
CD25-PE
Foxp3-Alexa488
Foxp3-Alexa488
Foxp3-Alexa488
