# Supplementary material for: Effect of 12-O-tetradecanoylphorbol-13-acetate-induced psoriasis-like skin lesions on systemic inflammation and atherosclerosis in hypercholesterolaemic apolipoprotein E deficient mice
Source: BMC Dermatol. 2016 Jul 11;16:9. doi: 10.1186/s12895-016-0046-1 (PMC4940745; doi:10.1186/s12895-016-0046-1)
Supplement: Additional file 4: — Mouse body weight and plasma cholesterol. Mean ± SEM, unpaired parametric test control vs. 12-O-tetradecanoylphorbol-13-acetate (TPA) mice at baseline and termination in both studies (no statistical significant differences were found between the two groups of mice). (DOCX 14 kb) [file 12895_2016_46_MOESM4_ESM.docx]

**Additional file 4. Mouse body weight and plasma cholesterol.**

|  |  | **Study 1** | | **Study 2** | |
| --- | --- | --- | --- | --- | --- |
|  |  | Control (n=5) | TPA (n=7) | Control (n=15) | TPA (n=15) |
| **Mouse weight (g)** | Baseline | 21.4 ±1.2 | 21.8 ±0.6 | 23.6 ±0.3 | 23.1 ±0.2 |
|  | Termination | 25.2 ±2.1 | 25.2 ±0.7 | 26.6 ±0.4 | 25.8 ±0.3 |
| **Total cholesterol (mM)** | Baseline | 16.0 ±2.5 | 14.8 ±0.6 | 10.8 ±0.4 | 10.4 ±0.4 |
|  | Termination | 11.3 ±0.5 | 13.5 ±0.9 | 11.6 ±0.5 | 10.6 ±0.5 |

Mean ±SEM, unpaired parametric test control vs. 12-*O*-tetradecanoylphorbol-13-acetate (TPA) mice at baseline and termination in both studies (no statistical significant differences were found between the two groups of mice).
